# Fungal Exosome-Like Nanoparticles from Huaier (Trametes robiniophila Murr.) Exhibit Antibreast Cancer Activity

**DOI:** 10.1016/j.cdnut.2026.107657

**Published:** 2026-02-23

**Authors:** Hua Zhang, Xiao Y Liang, Jie Yuan, Hua Kang

**Affiliations:** 1 Center for Thyroid and Breast Surgery, Department of General Surgery, Xuanwu Hospital, Capital Medical University, Beijing, PR China; 2 Department of General Surgery, Taihe Hospital, Hubei University of Medicine, Shiyan, PR China; 3 Department of Thoracic and Cardiac Surgery, Taihe Hospital, Hubei Medical University, Shiyan, PR China

**Keywords:** breast cancer, Huaier, exosome-like nanoparticles, anticancer mechanisms, miRNA

## Abstract

**Background:**

Breast cancer (BC) is a leading cause of morbidity and mortality among females globally. Despite advances in treatment, the disease remains a significant public health challenge. Recently, exosomes and exosome-like nanoparticles (ELNs) derived from various organisms have emerged as potential therapeutic agents.

**Objectives:**

This study aimed to investigate the effects of Huaier (*Trametes robiniophila* Murr.)-derived ELNs on the viability and behavior of human BC cells and to explore the underlying mechanisms, including the role of microRNAs (miRNAs).

**Methods:**

Huaier ELNs were isolated and purified from Huaier through differential ultracentrifugation. *In vitro* assays, including Cell Counting Kit-8, colony formation, flow cytometry, transwell invasion, and wound healing assays, were used to assess cell viability, migration, and invasion. *In vivo*, a murine model was employed to study tumor growth. RNA sequencing, miRNA sequencing, quantitative polymerase chain reaction, and dual-luciferase reporter assays were performed to identify the molecular mechanisms, particularly focusing on oncogenic signaling pathways and miRNA involvement.

**Results:**

Huaier ELNs significantly suppressed BC cell proliferation, migration, and invasion *in vitro*. *In vivo*, they resulted in substantial inhibition of tumor growth. RNA and miRNA sequencing revealed that Huaier ELNs modulated the expression of multiple genes associated with oncogenic pathways, and miRNAs enriched in the ELNs played key roles in a cross-kingdom regulatory mechanism.

**Conclusions:**

Huaier-derived ELNs exhibit potential as a therapeutic agent for BC, showing significant antitumor effects both *in vitro* and *in vivo*. The study highlighted the involvement of miRNAs in the regulation of oncogenic signaling pathways, offering a new perspective for therapeutic strategies targeting BC.

## Introduction

Breast cancer (BC), the leading cause of cancer-related mortality among females, places a substantial disease burden on the female population all over the world [1]. Although conventional treatments like mastectomy [2], chemoradiotherapy [3], and targeted therapies [4] have improved survival rates, they are often associated with severe complications, including cardiac toxicity, which has become the second leading cause of mortality among patients with BC [5]. Therefore, the development of novel therapeutic strategies with improved safety profiles remains an urgent, unmet need. In this context, natural products, particularly those derived from edible sources, offer a promising avenue for complementary and alternative interventions due to their favorable safety and biocompatibility profiles.

Exosomes and exosome-like nanoparticles (ELNs), nanosized lipid-bilayer vesicles which are secreted by virtually all cell types of the organisms and carry diverse bioactive cargo including nucleic acids, lipids, and proteins, have emerged as crucial mediators of intercellular communication [6], facilitating material transfer, information exchange, and immune regulation. In recent years, lots of studies associated with herb ELNs have gained significant attention in biomedical research due to their low immunogenicity, biocompatibility, stability, and biosafety [7,8]. The fungi ELNs were also investigated, and some of them have become promising candidates for therapeutic applications in anti-inflammatory, antitumor, and radioprotective interventions [9,10]. It demonstrated that ELNs derived from shiitake mushroom (Lentinula edodes) could protect against acute liver injury in mice by suppressing NOD-like receptor protein 3 (NLRP3) inflammasome activation and inflammatory protein expression [11]. The ELNs derived from Phellinus linteus could inhibit UV-induced skin aging by repressing microtubule-associated monooxygenase, calponin and LIM domain containing 2 expression through cross-kingdom regulation [12], and suppress metastatic hepatocellular carcinoma by reactive oxygen species generation and microbiota rebalancing [13]. The ELNs of Biyang floral mushrooms showed promise as a novel and natural radioprotective agent for preventing oxidative stress damage induced by ionizing radiation [9]. The ELNs from Cordyceps militaris potentiate immunomodulatory and antitumor effects by reprogramming macrophages [14]. Poria cocos-derived ELNs alleviate metabolic dysfunction-associated fatty liver disease by promoting mitophagy and inhibiting NLRP3 inflammasome activation [15].

Huaier (Trametes robiniophila Murr.), a traditional Chinese medicinal fungus belonging to Basidiomycota, which contains abundant proteoglycans, polysaccharides, ketones, and alkaloids, has shown therapeutic potential in various diseases [[16], [17], [18], [19]]. Multiple studies indicated that Huaier could be used to treat BC [20,21]. However, the ELNs of Huaier have never been investigated.

In the current study, the Huaier ELNs were first isolated and identified successfully by using differential centrifugations and ultracentrifugations. The internalization of Huaier ELNs by human BC cells [Michigan Cancer Foundation-7 (MCF-7)] negatively affects their viability, suggesting the anticancer properties of Huaier ELNs. Furthermore, a series of experiments, including flow cytometric detection, transwell invasion, and a xenograft mouse model, RNA sequencing (RNA-seq), were implemented.

## Methods

### Isolation and purification of ELNs

**FIGURE 1:**
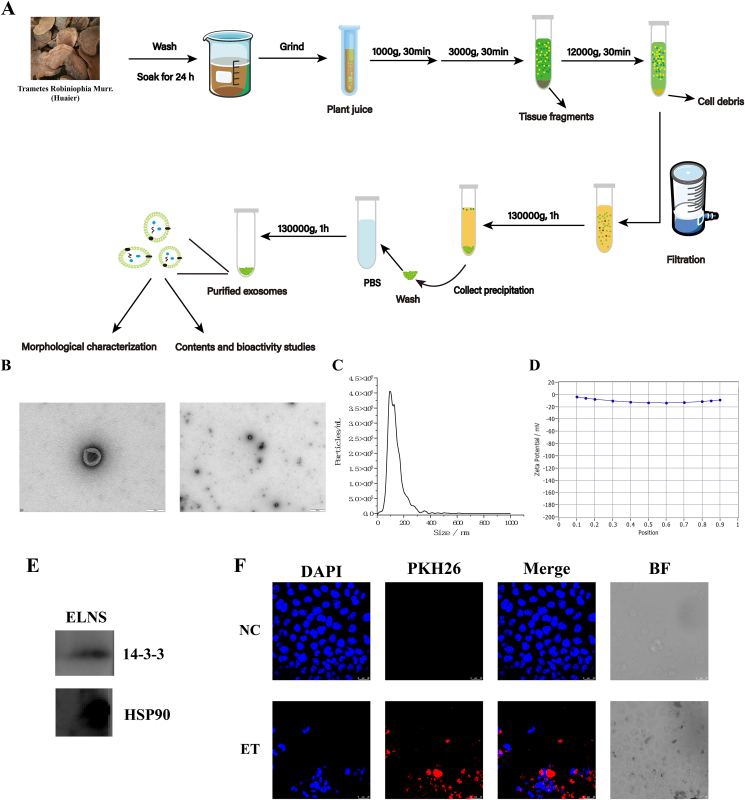
Isolation, characterization, and internalization of Huaier ELNs. (A) Schematic illustration of the process for isolating and purifying ELNs. (B) TEM image of ELNs. Scale bar = 100 nm. (C) Graphical analysis of the size distribution and concentration of ELNs. (D) The zeta potential of ELNs was measured by ZetaView. (E) ELNs validation by western blotting, indicating the 14-3-3 and HSP70 markers for fungal ELNs. (F) Confocal microscopy image showing the internalization of ELNs by MCF-7 cells. ELNs are labeled with PKH26 (red), and nuclei are stained with DAPI (blue). Scale bar = 25 μm. BF, Brightfield; DAPI, 4',6-diamidino-2-phenylindole; ELNs, exosome-like nanoparticles; ET, ELNs-treated; HSP70, heat shock protein 70; MCF-7, Michigan Cancer Foundation-7; NC, normal control; PBS, phosphate-buffered saline; TEM, transmission electron microscopy.

The ELNs were isolated from the juice of Huaier using a series of differential centrifugations and ultracentrifugations (Figure 1A). Briefly, 10 g of dry Huaier was soaked in phosphate-buffered saline (PBS) buffer for 24 h at 4°C, and ground thoroughly. The solution was filtered through sterile gauze to obtain the leaching filtrate. The filtrate was sequentially centrifuged at 1000 × *g*, 3,000 × *g*, and 12,000 × *g* for 30 min at 4°C, respectively, followed by filtration through a 0.22 μm membrane. The supernatant was then ultracentrifuged at 130,000 × *g* for 1 h at 4°C, and the resulting pellet was resuspended in PBS and subjected to a second ultracentrifugation under identical conditions. Finally, purified ELNs were resuspended in PBS and stored at –80°C for further experiments.

### Characterization of ELNs

The ELNs were characterized using nanoparticle tracking analysis (NTA) and transmission electron microscopy (TEM). For NTA, samples were diluted in PBS and analyzed using a ZetaView system (Particle Metrix) to determine particle size distribution and concentration. For TEM analysis, ELNs were negatively stained with 2% uranyl acetate and imaged using a Hitachi HT-7700 microscope (Hitachi) at 80 kV.

### Western blotting

According to the previous studies [22,23, 2[22,23], 2 conserved proteins [14-3-3 and heat shock protein 70 (HSP70)] were used as the markers of the fungal ELNs. Huaier ELNs proteins were separated by denaturing SDS-PAGE and subsequently transferred onto a polyvinylidene difluoride membrane. The membrane was blocked with 5% skimmed milk powder in PBS containing Tween 20 (PBST) for 1 h, followed by washing with PBST. It was then incubated with primary antibodies against 14-3-3 and HSP70 (Proteintech) according to the manufacturer’s instructions. Afterward, the membrane was incubated with a corresponding secondary antibody for 1 h and washed again. Finally, protein bands were visualized by incubating the membrane with SuperSignal West Pico Western HRP substrate for 3 min at room temperature, and images were captured using a Tanon 5200 Multi-imaging system (Tanon).

### Cell culture

The BC cell lines [MCF-7, MD Anderson - Metastatic Breast (MDA-MB)-231, and MDA-MB-436] were obtained from Procell. They were maintained in Dulbecco’s modified Eagle medium supplemented with 10% fetal bovine serum (FBS) and 1% penicillin/streptomycin at 37°C in a humidified 5% CO_2_ atmosphere.

### Stability evaluation of Huaier ELNs *in vitro*

The particle size and concentration changes of Huaier ELNs after incubation with various biological lipids were assessed using NTA. Specifically, 5 μL of ELNs was combined with 45 μL of PBS, 10% FBS, simulated intestinal fluid (SIF), or simulated gastric fluid (SGF), and incubated at 37°C for 0, 2, 4, and 8 h. After incubation, each mixture was diluted to an appropriate concentration, and NTA was used to determine changes in mean particle size and size distribution.

### Cell internalization

The ELNs were labeled with PKH26 red fluorescent dye (Sigma-Aldrich) according to the manufacturer’s protocol. Briefly, 50 μL of ELNs was mixed with 4 μL PKH26 in 2 mL PBS and incubated for 5 min at room temperature. The reaction was stopped by adding 2 mL FBS, followed by ultracentrifugation at 100,000 × *g* for 90 min at 4°C. Labeled ELNs were resuspended in PBS and incubated with MCF-7 cells for 24 h. Cells were fixed with 4% paraformaldehyde, stained with 4',6-diamidino-2-phenylindole (DAPI), and imaged using confocal microscopy (CM).

In addition, ELNs were labeled with carboxyfluorescein diacetate succinimidyl ester (CFDA-SE) (Solarbio). Briefly, reconstituted CFDA-SE (1 mM stock) was incubated with ELNs at a final concentration of 0.02 μM for 15 min at 37°C in the dark. The reaction was stopped with PBS containing 10% FBS. Labeled ELNs were washed by ultracentrifugation (100,000 × *g*, 1 h, 4°C) and resuspended in PBS, incubated with MCF-7 cells for 24 h. To verify intracellular localization, sorted cells were counterstained with DAPI and imaged with a CM.

### Cell proliferation assay

Cell viability was assessed using the Cell Counting Kit-8 (CCK-8) assay (Solarbio). The BC cells (MCF-7, MDA-MB-231, and MDA-MB-436) were seeded in 96-well plates and treated with PBS [normal control (NC) group] or ELNs [ELNs-treated (ET) group] for 0, 24, 48, and 72 h. After treatment, 10 μL CCK-8 reagent was added to each well and incubated for 1 h. Absorbance was measured at 450 nm using a microplate reader.

### Cell clone formation assay

The BC cells were plated into 6-well plates and cultured for 10–14 d. Subsequently, they were digested at the logarithmic phase to produce a single-cell suspension using culture medium. Cells were stained with 0.4% crystal violet (Solarbio), and the number of colonies was calculated under an inverted microscope (Leica) finally.

### Apoptosis analysis

Apoptosis was assessed using Annexin-V–fluorescein isothiocyanate (FITC)/propidium iodide staining (Solarbio). The BC cells (MCF-7, MDA-MB-231, and MDA-MB-436) were treated with PBS or ELNs for 48 h, harvested, and stained according to the manufacturer’s protocol. Flow cytometry analysis was performed using a CytoFLEX S flow cytometer (Beckman Coulter).

### Wound healing assay

The BC cells (MCF-7, MDA-MB-231 and MDA-MB-436) were seeded in 6-well plates. After 12 h, the confluent monolayers in each well were washed with PBS, and a wound was generated with a 200 μL sterile pipette tip. Wound healing was measured, and photographed images were taken at 200 magnification using a Zeiss microscope (Leica) from each well at 0, 24, and 48 h postinjury time points after the wound was created.

### Transwell migration assay

The BC cells in serum-free medium were seeded in the upper chamber of 24-well transwell plates (Corning), with PBS or ELNs treatment. The lower chamber contained complete medium. After 24 h, migrated cells were fixed, stained with crystal violet, and quantified using ImageJ.

### RNA extraction

The total RNAs were extracted from all samples using TRIzol reagent (Invitrogen) following the manufacturer’s protocol. Briefly, samples were homogenized in TRIzol and incubated for 5 min at room temperature. Chloroform was added, and the mixture was centrifuged at 12,000 × *g* for 15 min at 4°C. The aqueous phase was collected, and RNA was precipitated with isopropanol. RNA pellets were washed with 75% ethanol and resuspended in diethylpyrocarbonate -treated water. RNA concentration and integrity were assessed using the Agilent 2100/4200 Bioanalyzer System (Agilent Technologies).

### RNA-seq and analyses

The cDNA libraries were constructed using the total RNAs with RNA-seq Library Prep Kit (Illumina) according to the instructions of the manufacturer. These libraries were sequenced with Illumina HiseqTM sequence platform. Using the paired-end RNA-seq approach, 150 bp paired-end raw reads would be generated.

Before mapping, these low-quality reads (1, reads containing sequencing adaptors; 2, reads containing sequencing primers; 3, nucleotides with Q quality score lower than 20) were discarded. The obtained high-quality clean reads were mapped to the human genome with HISAT2 software [24]. Only mapped reads with 1 genomic location were used for further analysis. To evaluate the expression level of genes, the fragments per kilobase million method was used, and the DESeq2 software [25] were applied to explore differentially expressed genes (DEG) with fold change (FC) (≥2 or ≤0.5) and false discovery rate (FDR) (≤0.01) cutoffs were used.

To analyze the gene function and frequency distribution of functional categories, Gene Ontology (GO) and Kyoto Encyclopedia of Genes and Genomes (KEGG) analysis were performed using the Driver and Vehicle Information Database bioinformatics database [26]. Networks were constructed by analyzing the Pearson’s correlation coefficient for the expression levels of genes, and the Cytoscape (version 3.0.2) was applied to display the coexpression network [27].

### MiRNA sequencing and analyses

Total RNAs (3 μg) from each sample were used for small RNA cDNA library preparation with NEBNext Multiplex Small RNA Library Prep Set for Illumina (NEB), and the generated libraries were sequenced on a Novaseq 6000 platform. To ensure the quality of further analysis and obtain clean reads, the raw reads were filtered with custom Perl and Python scripts; thus, some low-quality reads were removed.

The high-quality reads (18–30 bp) were mapped to their reference genome and analyzed with the bowtie package. These successfully mapped reads were aligned with the miRBase22.0 database for the identification of known miRNA. MirDeep2’s quantifier.pl was used to obtain the miRNA counts. Custom scripts were applied to obtain base bias on the first position of identified miRNA with a certain length and each position of all the miRNA identified, respectively.

The expression levels of miRNAs were measured by transcripts per million through the normalization formula. The human genome was used as a reference genome for predicting the target genes with Miranda3.3 software, which is based on a comparison of miRNAs' complementarity to 3' untranslated region (UTR) regions. The binding energy of the duplex structure, evolutionary conservation of the whole target site and its position within 3' UTR are calculated and account for a final result, which is a weighted sum of match and mismatch scores for base pairs and gap penalties.

### Real-time PCR

Real-time qPCR experiments were performed for detecting the expression levels of human mRNAs, and some specific primers were designed for qPCR accordingly. The primer sequences were as follows: CD24: 5'-CAC TGC TCC TAC CCA CGC A-3' (forward), 5'- TGG TGG TGG CAT TAG TTG GAT T-3' (reverse); CD36: 5'-TTT CCT GCA GAA TAC CAT TTG ATC C-3' (forward), 5'-TCT ACA AGC TCT GGT TCT TAT TCAC-3' (reverse); FOSL: 5'-ATC ACT GCC ACA CTC TCC AT-3' (forward), 5'-GGG CTG GTG AGT TAG TGT TC-3' (reverse); HTRA: 5'-GTC ATT GGC ATC AAC ACG CT-3' (forward), 5'-CTG CTC TCA ATG AAC TGC CA-3' (reverse); GAPDH: 5'-GGA GTC CAC TGG CGT CTT CA-3' (forward), 5'-GTC ATG AGT CCT TCC ACG ATA CC- 3' (reverse). The cDNA synthesis was conducted by standard procedures, and real-time PCR was implemented on the BioRad S1000 (BioRad) with Bestar SYBR Green RT-PCR Master Mix (DBI Bioscience). The PCR conditions consist of denaturing at 95˚C for 8 min, 35 cycles of denaturing at 95˚C for 15 s, annealing, and extension at 60˚C for 1 min. The qPCR amplifications were performed in triplicate for each sample. Transcript levels for the genes were measured in comparison with the housekeeping gene GAPDH as an internal reference standard, using the 2-ΔΔCT method [28].

### Dual-luciferase reporter gene assay

The synthetic myristoylated alanine-rich C-kinase substrate (MARCKS) 3' UTR fragment was introduced into pMIR-reporter (Huayuyang) to design complementary mutation sites of seed sequence on MARCKS wild type (WT). After digestion, the target fragment was inserted into the pMIR-reporter plasmid with T4 DNA ligase. The correctly sequenced luciferase reporter plasmids WT and mutant type were cotransfected with Huaier miRNA474a into HEK-293T cells. After 48 h of transfection, the cells were harvested and lysed. A luciferase assay kit (Beyotime) and a Glomax 20/20 luminometer fluorescence detector (Promega) were used to detect luciferase activity. The experiment in each group was repeated 5 times.

### Xenograft mouse model

All animal experiments were carried out in the animal laboratory center of Hubei University of Medicine in accordance with the Guide for the Care and Use of Laboratory Animals. A total of 12 male athymic Bagg Albino/c (BALB/c) nude mice (4–5 wk old) were used. In brief, 10^6^ cells (MCF-7) were subcutaneously injected into each mouse. When the tumor reached 100 mm^3^, these mice were randomly divided into NC and ET groups, and each group contained 6 mice.

The mice from the NC or ET groups were injected with PBS (100 μL) or ELNs (100 μL, 1010 particles/mL) by tail every 2 d, respectively. After 5 wk of injection, the mice were killed, and the tumors were carefully removed and photographed. The parts of the organs were fixed in paraformaldehyde solution (4%, v/v) and embedded in paraffin. The tissues were sectioned (5μm) and stained with hematoxylin and eosin (HE).

### Statistical analysis

All statistical analyses were performed using GraphPad Prism (v9.0) and Microsoft Excel (2016). Data are presented as mean ± SD. Comparisons between 2 groups were analyzed using Student’s *t*-test, with *P* < 0.05 considered statistically significant.

## Results

### Isolation, characterization, and internalization of ELNs

The ELNs of Huaier were successfully isolated using a combination of filtration and differential ultracentrifugation (Figure 1A). TEM images revealed that the isolated particles exhibited characteristic cup-shaped morphology with a typical exosome-like structure (Figure 1B). NTA results demonstrated that the purified ELNs had an median diameter of 121.3 nm (Figure 1C), and the yield of ELNs extracted from Huaier was 1.2 × 10^10^ particles/g. Moreover, ELNs had a zeta potential value of –6.90 mv (Figure 1D) and were validated by Western blotting using markers (14-3-3 and HSP70) expressed on their outer membrane (Figure 1E).

To investigate the cellular uptake of ELNs, MCF-7 cells were treated with the ELNs, which were labeled with PKH26 and CFDA-SE. The CM analysis revealed distinct red or green fluorescence in ET cells (ET group), indicating successful internalization, whereas no fluorescence was observed in NC cells (NC group) (Figure 1F and Supplemental Figure 1). These findings demonstrate that ELNs can be effectively internalized by BC cells.

### Huaier ELNs regulate proliferation and apoptosis of BC cells

**FIGURE 2:**
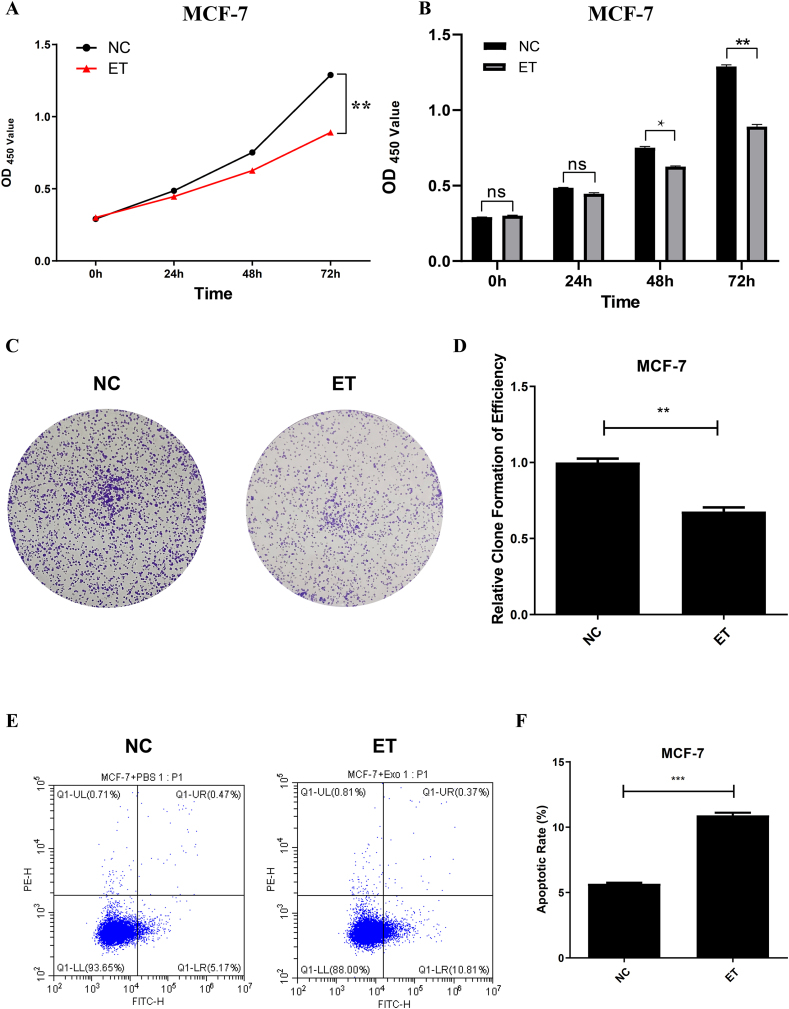
Huaier ELNs regulate the proliferation and apoptosis of BC cells. (A, B) Proliferation of MCF-7 cells in PBS–treated (NC) and ET groups was detected by CCK-8 assays. (C, D) Representative and quantitative images of clone formation assays. (E, F) Flow cytometric analysis of apoptosis in MCF-7 cells from NC and ET groups. ∗*P* < 0.05, ∗∗*P* < 0.01, ∗∗∗*P* < 0.001. BC, breast cancer; CCK-8, Cell Counting Kit-8; ELNs, exosome-like nanoparticles; ET, ELNs-treated; OD, optical density; MCF-7, Michigan Cancer Foundation-7; NC, normal control; PBS, phosphate-buffered saline.

Because MCF-7 cells were shown to internalize Huaier ELNs, we decided to assess whether ELNs affect the viability of the cells. The MCF-7 cells were treated with PBS and Huaier ELNs. The concentration of ELNs in the culture medium was 10^9^ particles/mL. A cell viability assay for both the PBS (NC) and ET cells in 96-well dishes after various periods of plating (0, 24, 48, and 72 h) was conducted. The quantification revealed that ELNs-treated cells showed significantly reduced absorbance values at all the time points, indicating that the BC cells proliferate at a much slower rate on ELNs treatment (*P* < 0.01) (Figure 2A, B). In addition, the cell clone formation assay was conducted, and the results also indicated that ELNs treatment could markedly inhibit the proliferation of BC cells (*P* < 0.01) (Figure 2C, D).

To decipher whether ELNs treatment also induced cell death, we tried to count the number of apoptotic cells in both PBS and ELNs-treatment cells. We stained both cultures with Annexin-V conjugated with FITC, and the number of labeled cells was determined using flow cytometry-based methods. The results indicated that a significant increase in the number of Annexin-FITC labeled cells on ELNs-treatment compared with PBS-treated cells, indicating a large elevation in the number of apoptotic cells (Figure 2E, F).

To further verify this result, we also performed similar experiments, including CCK-8, clone formation assay, and flow cytometric detection in 2 other BC cell lines (MDA-MB-231 and MDA-MB-436). The results were consistent with those in MCF-7 cells (Supplemental Figures 2 and 3). Hence, all results indicated that Huaier ELNs repress proliferation and promote apoptosis of the BC cells.

### Huaier ELNs regulate the migratory potential of BC cells

**FIGURE 3:**
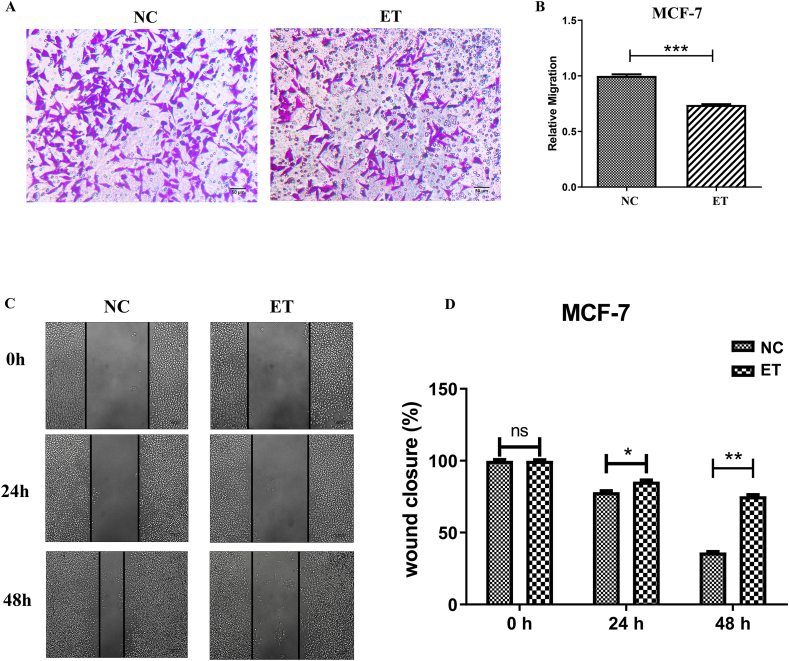
Huaier ELNs regulate the migration of BC cells. (A, B) Representative and quantitative images of transwell migration assays in MCF-7 cells of NC and ET groups. (C, D) Representative and quantitative images of wound healing assays in MCF-7 cells from NC and ET groups. ∗*P* < 0.05, ∗∗*P* < 0.01, ∗∗∗*P* < 0.001. BC, breast cancer; ELNs, exosome-like nanoparticles; ET, ELNs-treated; MCF-7, Michigan Cancer Foundation-7; NC, normal control.

Subsequently, we tended to explore whether Huaier ELNs can regulate the migratory potential of the BC cells, so the transwell migration assay was carried out. In this assay, 2 chambers were separated by an 8 μm pore transwell chamber. About 10^5^ cells (MCF-7) were first placed on the Matrigel-coated upper chamber, and the number of the invaded cells that reached the other chamber was fixed and counted. The results indicated a significant reduction in the number of invading cells on ELNs-treatment (ET) compared with control (NC) cells, suggesting that Huaier ELNs treatment could impede the ability of BC cells to migrate (*P* < 0.01) (Figure 3A, B). To further estimate the migration effect of this ELNs on BC cells, wound healing assay was performed, and the results showed that Huaier ELNs repressed cell invasion posttreatment for 24 and 48 h, compared with the control (*P* < 0.01) (Figure 3C, D).

Furthermore, similar experiments (transwell migration assay and wound healing assay) were also implemented in 2 other BC cell lines (MDA-MB-231 and MDA-MB-436). According to the analogous experimental results (Supplemental Figures 4 and 5), it is further indicated that Huaier ELNs inhibit the migration of BC cells.

### Huaier ELNs repress the development of tumor *in vivo*

**FIGURE 4:**
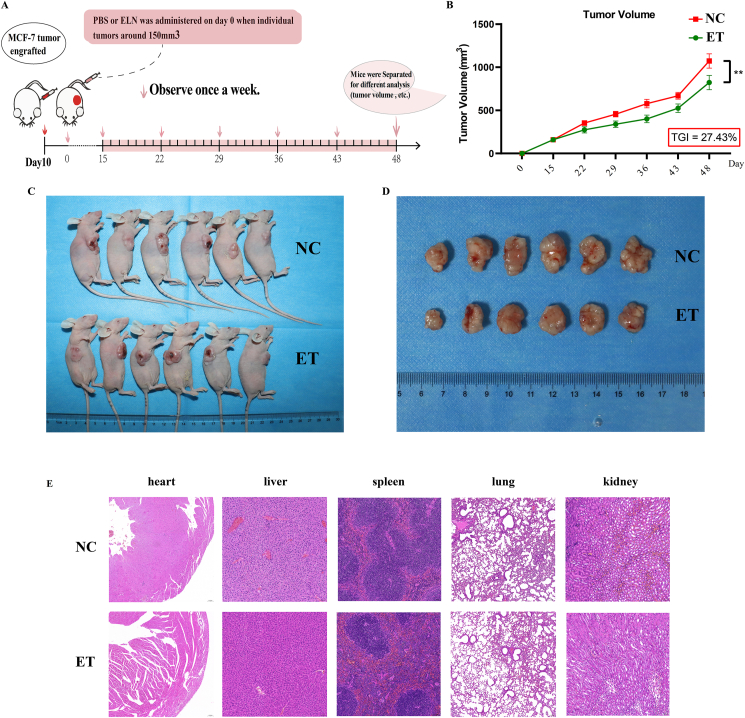
Huaier ELNs repress the development of tumors *in vivo*. (A) Experimental protocol schematic *in vivo*. (B) The mean tumor volumes of mice from PBS–treated NC and ET groups. (C, D) The morphological differences in tumor sizes of mice from NC and ET groups. (E) HE staining analysis for some organs (liver, lung, kidney, heart, and spleen) of the mice from the NC and ET groups. ∗*P* < 0.05, ∗∗*P* < 0.01, ∗∗∗*P* < 0.001. ELNs, exosome-like nanoparticles; ET, ELNs-treated; HE, hematoxylin and eosin; PBS, phosphate-buffered saline; NC, normal control; TGI, tumor growth inhibition.

To explore whether Huaier ELNs regulate the development of tumor *in vivo*, mice bearing subcutaneous breast tumors were established, and divided into NC (PBS-treated) and ET groups. Each of the mice from NC or ET groups was injected PBS (100 μL) or ELNs (100 μL; 10^10^ particles/mL) by tail every 2 d, respectively, and the body weights and tumor volumes of 2 groups were recorded during the treatment (Figure 4A), and the all mice did not exhibit any abnormalities during the entire treatment period.

Compared with the NC group, the mean tumor volumes in the ET group were reduced by 27.4% on day 48 (Figure 4B), which were consistent with morphological changes in tumor sizes (Figure 4C, D), indicating that ELNs-treatment could impede the development of the tumor *in vivo*. To assess the potential systemic toxicity of ELNs, we performed HE staining on major organs (liver, lung, kidney, heart, and spleen). The results showed no obvious histopathological differences between the NC and ET groups (Figure 4E), indicating that ELNs treatment did not induce any detectable histopathological alterations in these organs.

### Transcriptomic analysis of ET BC cells

**FIGURE 5:**
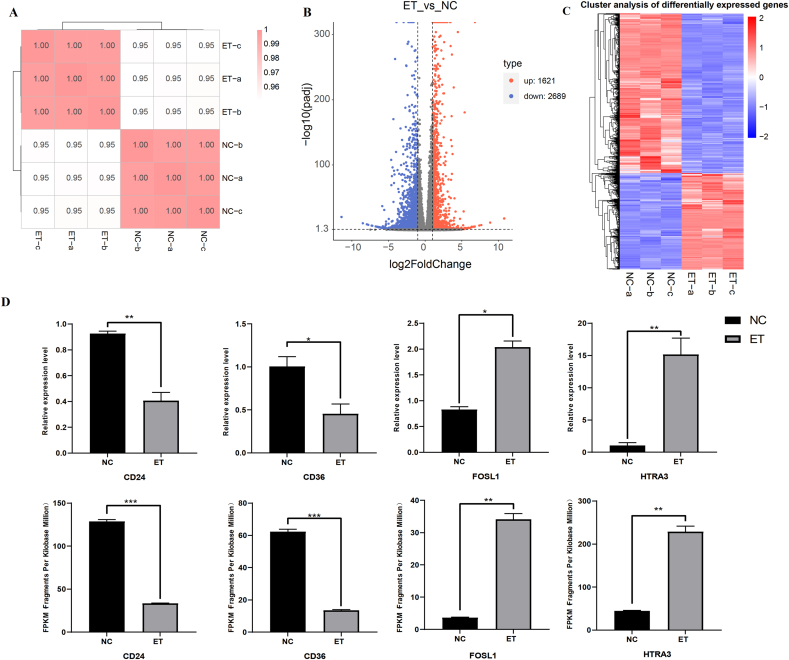
Deciphering the molecular mechanisms of Huaier ELNs function on cancer cells. (A) Heatmap clustering showing the sample correlation analysis of the samples from ET and NC groups. (B) Volcano plots indicate gene expression in ET vs. NC. The x-axis represents the log_2_ fold of changes, whereas the y-axis represents the –log10 significance of difference (*P* value). Genes with significant differential expression were shown in red (upregulated gene) or blue (downregulated gene), and genes that were not differentially expressed were shown in grey. (C) The heatmap for DEGs in ET vs. NC. (D) The mRNA expression levels of some DEGs were determined by high-throughput sequencing and RT-qPCR.∗*P* < 0.05, ∗∗*P* < 0.01, ∗∗∗*P* < 0.001. DEGs, differentially expressed genes; ELNs, exosome-like nanoparticles; ET, ELNs-treated; NC, normal control.

To explore the mechanisms behind the antitumorigenic functions of Huaier ELNs, we decided to conduct an RNA-seq analysis for the cell samples (MCF-7) with PBS (NC) or ELNs-treatment (ET). Six libraries (NC-a, NC-b, NC-c; ET-a, ET-b, and ET-c) were generated successfully, and subsequently experiments and analyses were carried out (Figure 5A).

There were 4310 DEGs found in ET compared with NC (FC ≤2 or ≤0.5 and *P* ≤ 0.05), with 1621 upregulated and 2689 downregulated ones (Supplemental Table 1 and Figure 5B, C). To validate the DEG results, we randomly selected 4 DEGs (CD24, CD36, FOSL, and HTRA) and performed RT-qPCR experiments for them. The results of RT-qPCR experiments were highly consistent with the results from RNA-seq (Figure 5D), suggesting that the RNA-seq data might be suitable for exploring the underlying mechanism.

**FIGURE 6:**
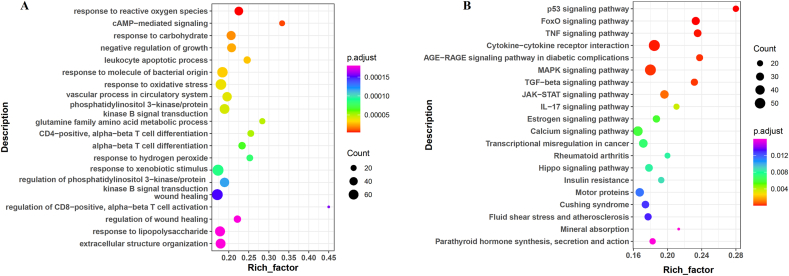
Functional analyses for genes affected by Huaier ELNs. (A) KEGG analyses of ET vs. NC groups from ET vs. NC groups. (B) GO analyses of DEGs from ET vs. NC groups. Only the top 20 terms are listed here. The rich factor reveals the enrichment degree of terms, whereas the vertical axis indicates the names of the enriched terms. The area of the node shows the number of genes, and the *P* value is demonstrated by a color scale with the statistical significance increasing from green to red. DEGs, differentially expressed genes; ELNs, exosome-like nanoparticles; ET, ELNs-treated; GO, Gene Ontology; KEGG, Kyoto Encyclopedia of Genes and Genomes; NC, normal control.

To identify the primary functions in which the DEGs are involved, KEGG analysis was performed. A total of 41 enriched pathways (*P* < 0.05, FDR <0.4) were identified (Supplemental Table 2 and Figure 6A). including several related to cancer progression and cell signaling, such as “p53 signaling pathway” (hsa04115), “Forkhead box class O (FoxO) signaling pathway” (hsa04068), and “TNF signaling pathway” (hsa04668). The upregulation of apoptosis-related pathways such as “Apoptosis” (hsa04210) and “Cytokine-cytokine receptor interaction” (hsa04060) was also observed, suggesting that Huaier ELNs may inhibit BC cell proliferation by modulating these critical cancer-related pathways (Supplemental Table 2 and Figure 6A). These findings highlight that Huaier ELNs primarily target pathways involved in tumorigenesis, immune modulation, and cell survival.

Subsequently, a total of 38 GO terms (*P* < 0.01, FDR <0.05) were identified (Supplemental Table 3). Multiple pathways were involved with cell state, signaling, and response to stress, such as “Response to oxidative stress” (GO:0006979), “Leukocyte apoptotic process” (GO:0071887), and “Negative regulation of growth” (GO:0045926) (Supplemental Table 3 and Figure 6B). These pathways further substantiate the anticancer potential of Huaier ELNs by promoting cell death and inhibiting BC cell proliferation.

### The miRNAs of Huaier ELNs and functional analyses

Among the biocargoes of ELNs, the miRNAs have attracted the most interest for functional and therapeutic importance. In this study, for the first time, the miRNAs of Huaier ELNs were identified by miRNA sequencing (miRNA-seq) experiment and analyses (Supplemental Table 4).

**FIGURE 7:**
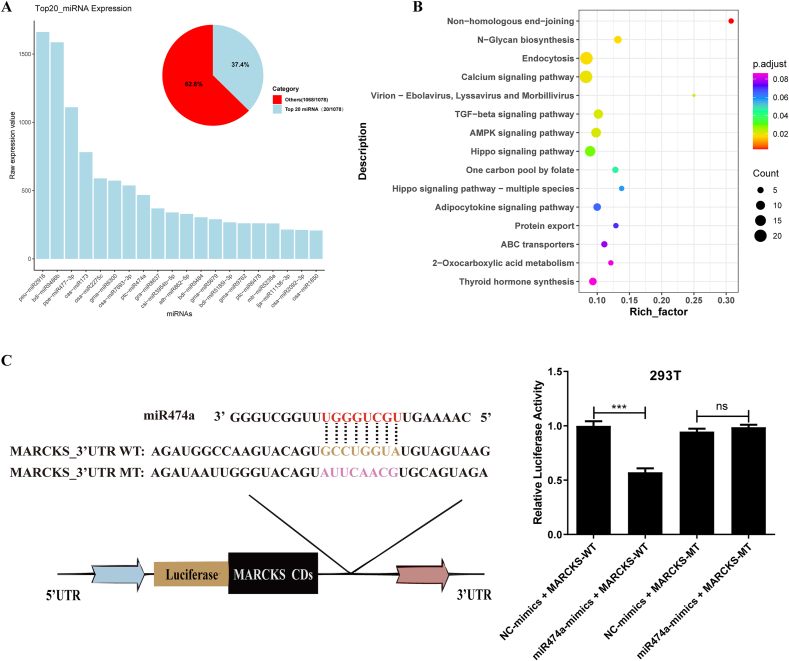
Identification and functional analyses of miRNAs of Huaier ELNs. (A) Identification of microRNAs (miRNAs) from Huaier ELNs by miRNA sequencing. (B) KEGG analyses of putative human target genes for 20 highly enriched miRNA in ELNs. Only the top 15 terms are listed here. (C) The interaction between miRNA474a highly enriched Huaier ELNs and its potential target gene (MARCKS) was validated by the dual-luciferase activity assay. ∗*P* < 0.05, ∗∗*P* < 0.01, ∗∗∗*P* < 0.001. ELNs, exosome-like nanoparticles; KEGG, Kyoto Encyclopedia of Genes and Genomes; MARCKS, myristoylated alanine-rich C-kinase substrate.

Moreover, the target genes of these highly enriched miRNAs (Top20) in Huaier ELNs were predicted (Figure 7A). GO analysis of predicted target genes revealed significant involvement in signal transduction pathways such as “Small GTPase-mediated signal transduction” (GO:0007264) and “Regulation of receptor-mediated endocytosis” (GO:0048259), both of which play pivotal roles in cancer progression and cell migration (Supplemental Table 5). Furthermore, KEGG pathway enrichment of miRNA target genes revealed several pathways relevant to cancer biology, including “Non-homologous end-joining” (hsa03450), “Endocytosis” (hsa04144), and “transforming growth factor (TGF)-β signaling pathway” (hsa04350) (Supplemental Table 6 and Figure 7B). These findings suggest that miRNAs within Huaier ELNs may regulate key cancer-related pathways involved in DNA repair, cell survival, and metastasis.

To further validate the prediction results, we performed a dual-luciferase assay to verify the interaction between miRNA474a and its potential target gene (MARCKS). This indicated that miRNA474a binding significantly reduced luciferase activity for the WT target gene, whereas binding to the mutant site did not affect luciferase activity (Figure 7C). Together, these results suggested that Huaier miRNAs have the potential to target human mRNAs.

## Discussion

Traditional medicinal resources have long been recognized as valuable therapeutic options for a wide range of diseases, including AIDS, dengue, rheumatoid arthritis, and cancer [29]. Huaier, a medicinal fungus with >1600 years of clinical application has been widely used as an adjuvant therapy in malignancies [20,30]. However, despite extensive studies focusing on Huaier-derived polysaccharides, proteoglycans, and small-molecule compounds, the specific bioactive constituents responsible for its anticancer effects remain incompletely understood. In the present study, the first systematic characterization of ELNs derived from Huaier was provided (Figure 1), and it indicated that Huaier ELNs could repress BC both *in vitro* and *in vivo* (FIGURE 2, FIGURE 3, FIGURE 4), but not induce injuries to normal cells and organs (Figure 4E and Supplemental Figure 6). These findings highlight that ELNs may represent a previously unrecognized and functionally significant bioactive fraction of Huaier, complementing and potentially surpassing traditional Huaier components in mechanistic specificity and delivery capability.

A substantial body of prior research has attributed Huaier’s antitumor properties primarily to its polysaccharides, proteins, or proteoglycan complexes. These components exert immunomodulatory effects, promote apoptosis, inhibit angiogenesis, or modulate signaling pathways such as phosphatidylinositol 3-kinase (PI3K)-Akt, mitogen-activated protein kinase (MAPK), and STAT3 [[17], [18], [19]]. However, these conventional components often face intrinsic limitations, including susceptibility to degradation, low targeting efficiency, and limited cellular uptake. In contrast, ELNs naturally possess a nanoscale lipid-bilayer structure capable of protecting internal biomolecules, such as miRNAs, lipids, and proteins from enzymatic digestion. This enables more efficient cellular internalization and delivery of bioactive cargo. In this study, to assess the potential of Huaier ELNs as orally delivered therapeutic or nutraceutical agents, ELNs were incubated in SGF and SIF at 37°C for ≤8 h. The NTA revealed that the mean particle size and size distribution of Huaier ELNs remained largely unchanged in both SIF and SGF over time (Supplemental Figure 7), supporting their suitability for oral administration and highlighting their relevance in nutritional and functional food applications.

Mechanistically, transcriptomic profiling revealed that Huaier ELNs profoundly reprogrammed cancer cell gene expression, with numerous DEGs enriched in pathways critically involved in oncogenesis. Notably, key pathways such as MAPK, PI3K-Akt, TGF-β, Janus Kinase–Signal Transducer and Activator of Transcription, FoxO, nuclear factor-κB, and Hippo signaling were significantly modulated. These pathways collectively regulate proliferation, apoptosis, migration, metabolism, and stress responses, providing a mechanistic basis for the observed biological effects. The parallel identification of highly enriched fungal miRNAs within Huaier ELNs, including miR2916, miR9486b, miR6300and miR474a, further supports a cross-kingdom regulatory mechanism. Dual-luciferase assays confirmed that miR474a directly targets human MARCKS, validating its functional capacity in mammalian cells. Together, these results align with emerging evidence showing that miRNAs from edible plants, fungi, and food-derived ELNs can survive digestion, enter circulation, and regulate mammalian gene expression [13,31,32]. The identification of functionally active fungal miRNAs in Huaier ELNs provides a novel mechanistic link between Huaier and its anticancer properties.

In addition to the anticancer effects observed in BC, our results may have broader implications across malignancies. The major pathways modulated by Huaier ELNs, particularly MAPK, TGF-β, PI3K-Akt, and Hippo, are conserved oncogenic networks implicated in hepatocellular carcinoma, gastric cancer, lung cancer, colorectal cancer, and sarcoma. Furthermore, ELNs from other fungi (e.g., Phellinus linteus, Lentinula edodes, Cordyceps militaris) have demonstrated anti-inflammatory or antitumor activities in hepatocellular carcinoma, colorectal cells, and UV-induced skin aging [[11], [12], [13], [14]]. These parallels reinforce the likelihood that Huaier ELNs possess therapeutic relevance beyond BC. Our findings therefore provide a compelling rationale for future evaluation of Huaier ELNs in additional tumor models, including triple-negative BC, hepatocellular carcinoma, gastric cancer, and colorectal cancer.

Collectively, this study positions Huaier ELNs as a previously overlooked yet highly potent bioactive component of Huaier. Their stability, efficient cellular uptake, multifaceted anticancer activity, and ability to regulate human gene expression through cross-kingdom fungal miRNAs suggest that ELNs may serve as next-generation Traditional Chinese Medicine (TCM)-derived nanotherapeutics. In conclusion, future work will focus on *1*) profiling the full molecular cargo of Huaier ELNs, *2*) exploring their pharmacokinetics and biodistribution, *3*) evaluating their effects across diverse cancer types, and *4*) investigating potential synergistic effects with chemotherapeutic agents or immunotherapies. In addition, the efficiency of BC cell uptake of exosomes will be further investigated, considering that PKH26, a lipophilic dye, can form fluorescent aggregates or micelles independent of the vesicles. A PKH26-only control processed through the same labeling and washing steps should be included to rule out potential artifacts. These steps will be crucial for advancing Huaier ELNs into clinically viable therapeutic strategies and expanding the landscape of natural ELNs-inspired nanomedicine.

## Author contributions

The authors’ responsibilities were as follows – HZ, HK: contributed to investigation, methodology, project administration, resources, software, visualization, writing–original draft, and writing–review & editing; XYL: contributed to conceptualization, data curation, investigation, project administration, and writing–original draft; JY: contributed to formal analysis, resources, software, supervision, validation, visualization, writing–original draft, and writing–review & editing; and all authors: read and approved the final manuscript.

## Data availability

The sequencing data generated in this study have been deposited in the National Center for Biotechnology Information under accession numbers PRJNA1265791 and PRJNA1288179.

## Funding

This work was supported by the Wu Jieping Medical Foundation (no. 320.6750.2023-18-8), Chen Xiaoping Medical Foundation (CXPJJH125004-132, CXPJJH125001-25127), and Key Medical Research Projects of Shiyan City (24Y045).

## Conflict of interest

The authors report no conflicts of interest.
