# Supplementary material for: Fungal Exosome-Like Nanoparticles from Huaier (Trametes robiniophila Murr.) Exhibit Antibreast Cancer Activity
Source: Curr Dev Nutr. 2026 Feb 23;10(4):107657. doi: 10.1016/j.cdnut.2026.107657 (PMC13015671; doi:10.1016/j.cdnut.2026.107657)
Supplement: Multimedia component 2 [file mmc2.pdf]

# Fungal Exosome-like Nanoparticles from Huaier (Trametes robiniophila Murr.) Exhibit Anti-Breast Cancer Activity

Hua Zhang, Xiaoyan Liang, Jie Yuan, Hua Kang*

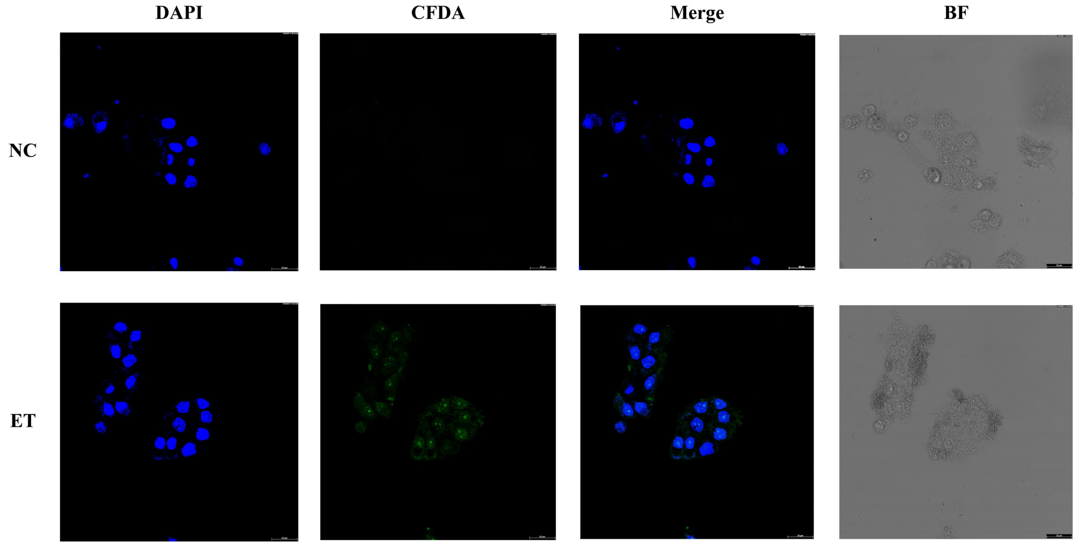


**Figure S1 Uptake of Huaier exosome-like nanoparticles (ELNs) by cells.** Representative fluorescence images of cells in the normal control (NC) and ELNs-treated (ET) groups. Nuclei were stained with DAPI (blue), and ELNs were labeled with CFDA (green). Merged images show intracellular uptake of CFDA-labeled ELNs in the ET group, whereas no green signal was detected in the NC group. BF, bright-field.

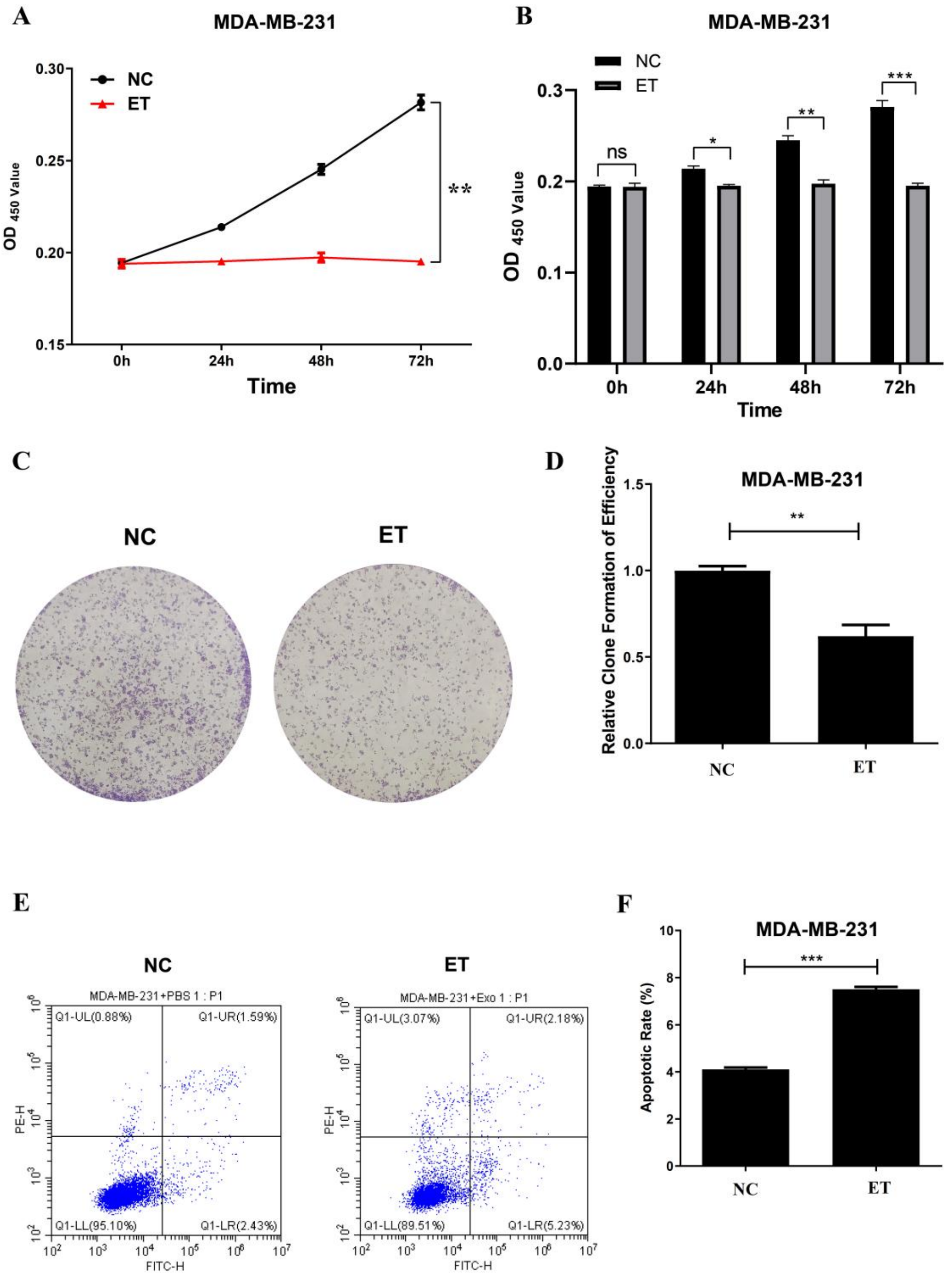


**Figure S2 Huaier exosome-like nanoparticles (ELNs) regulate proliferation and apoptosis of breast cancer (BC) cells.** (A) and (B) Proliferation of MDA-MB-231 cells in PBS-treated (normal control, NC) and ELNs-treated (ET) groups was detected by CCK-8 assays. (C) and (D) Representative and quantitative images of clone formation assays. (E) and (F) Flow cytometric analysis of apoptosis in MDA-MB-231 cells from NC and ET groups. *P<0.05, **P<0.01, ***P<0.001.

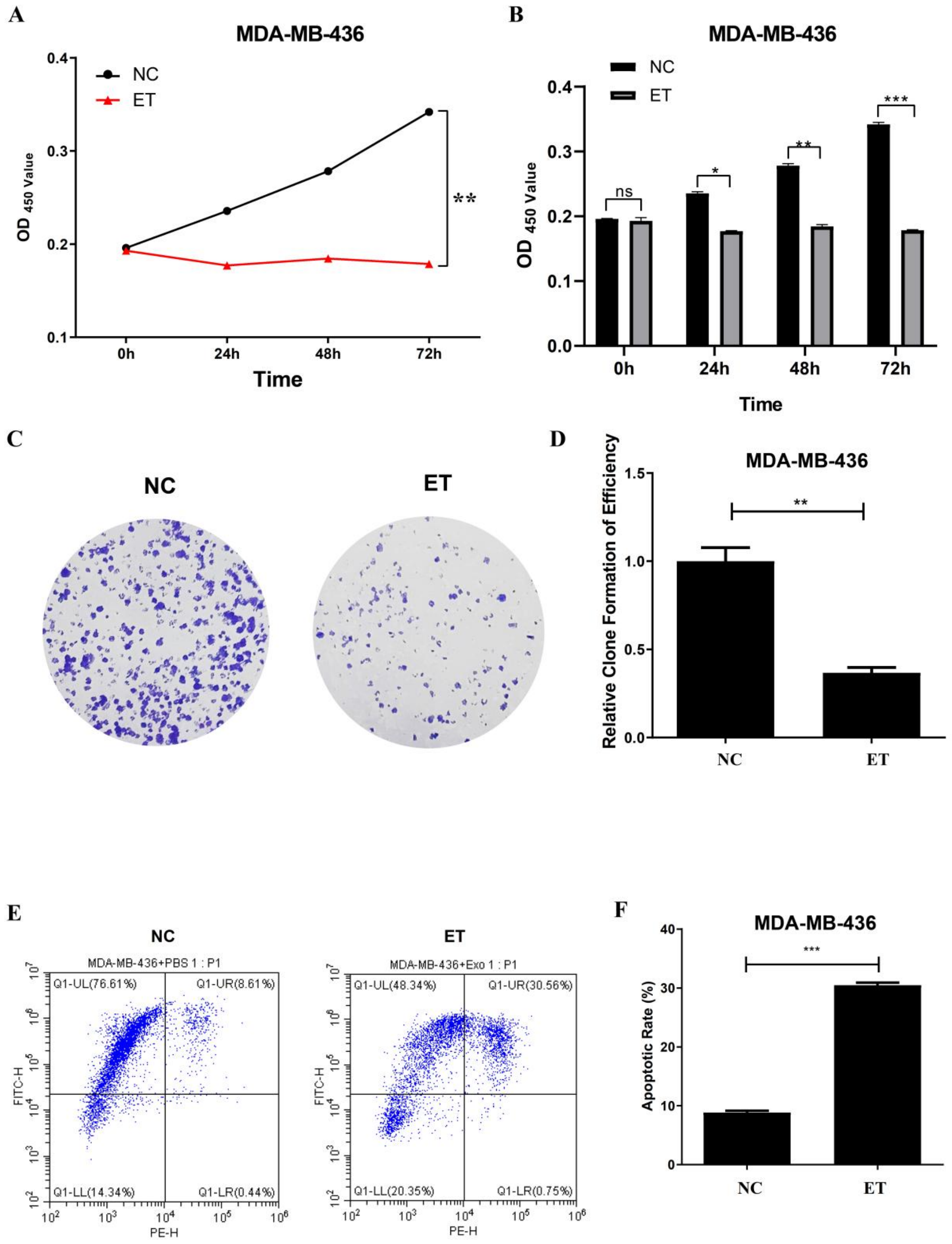


**Figure S3 Huaier exosome-like nanoparticles (ELNs) regulate proliferation and apoptosis of breast cancer (BC) cells.** (A) and (B) Proliferation of MDA-MB-436 cells in PBS-treated (normal control, NC) and ELNs-treated (ET) groups was detected by CCK-8 assays. (C) and (D) Representative and quantitative images of clone formation assays. (E) and (F) Flow cytometric analysis of apoptosis in MDA-MB-436 cells from NC and ET groups. *P<0.05, **P<0.01, ***P<0.001.

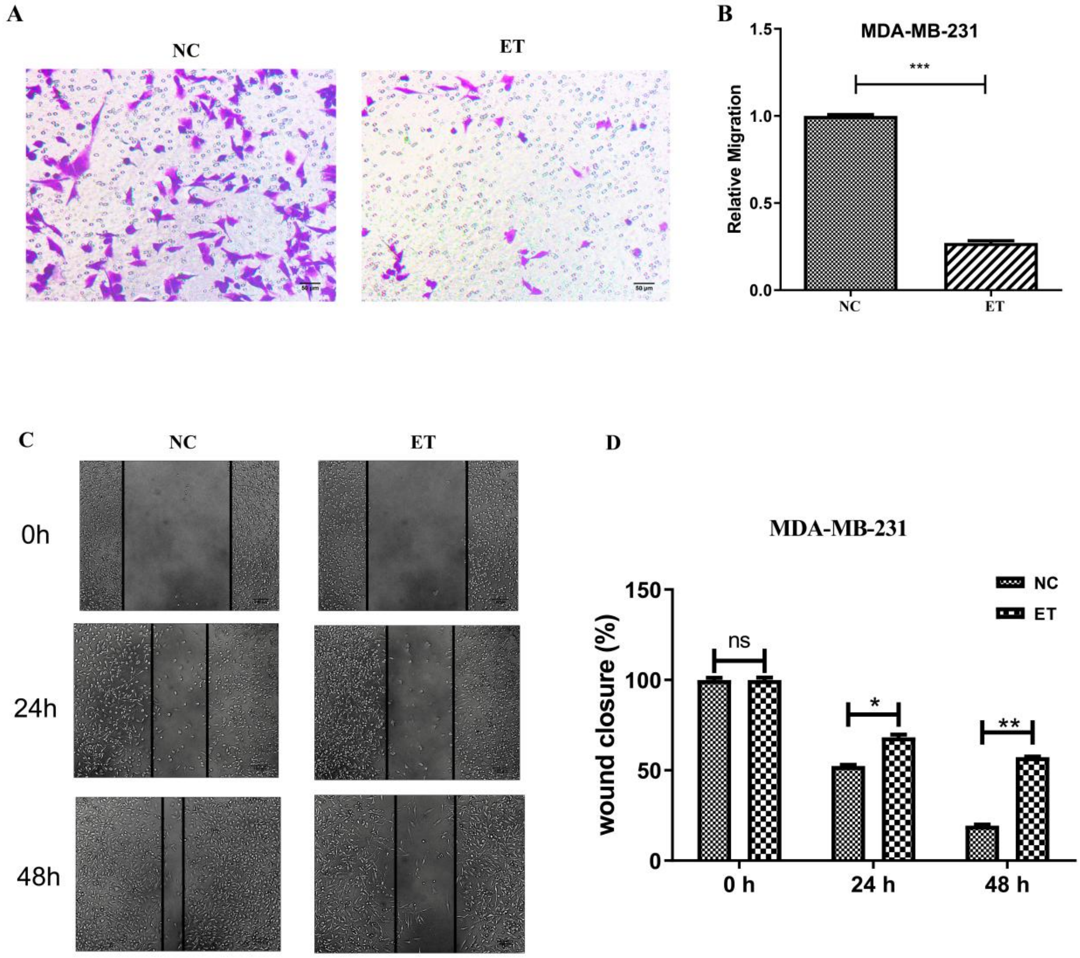


**Figure S4 Huaier exosome-like nanoparticles (ELNs) regulate migration of breast cancer(BC) cells.** (A) and (B) Representative and quantitative images of transwell migration assays in MDA-MB-231 cells of NC and ET groups. (C) and (D) Representative and quantitative images of wound healing assays in MDA-MB-231 cells from NC and ET groups. *P<0.05, **P<0.01, ***P<0.001.

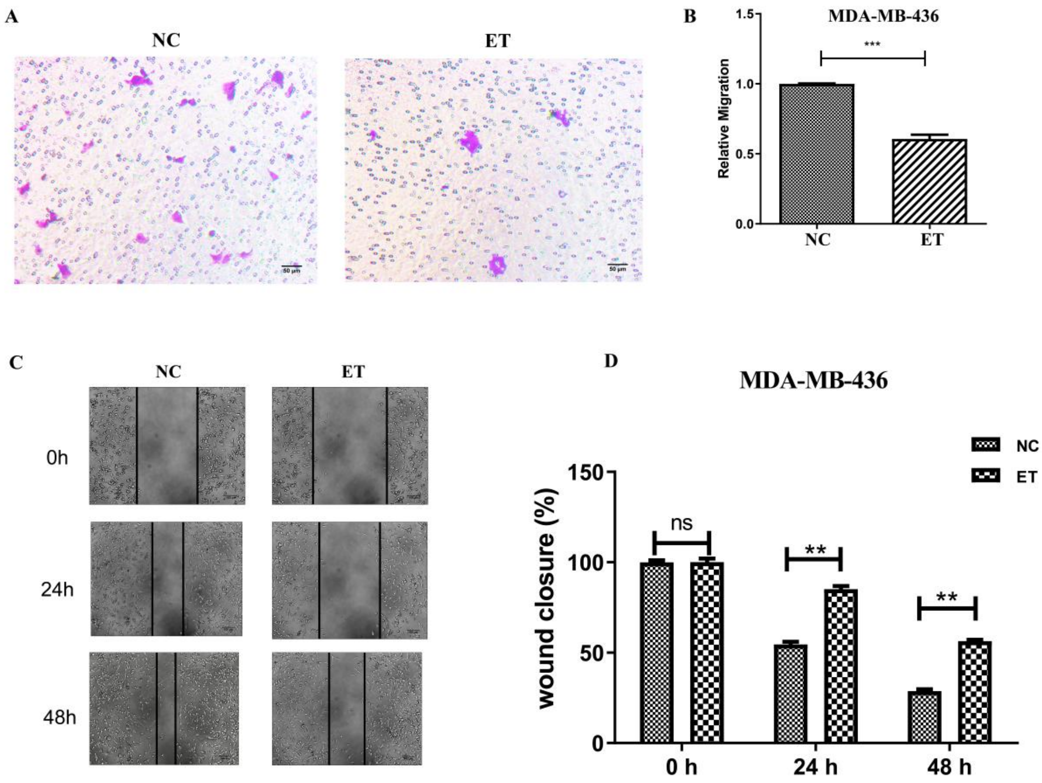


**Figure S5 Huaier exosome-like nanoparticles (ELNs) regulate migration of breast cancer(BC) cells.** (A) and (B) Representative and quantitative images of transwell migration assays in MDA-MB-436 cells of NC and ET groups. (C) and (D) Representative and quantitative images of wound healing assays in MDA-MB-436 cells from NC and ET groups. *P<0.05, **P<0.01, ***P<0.001.

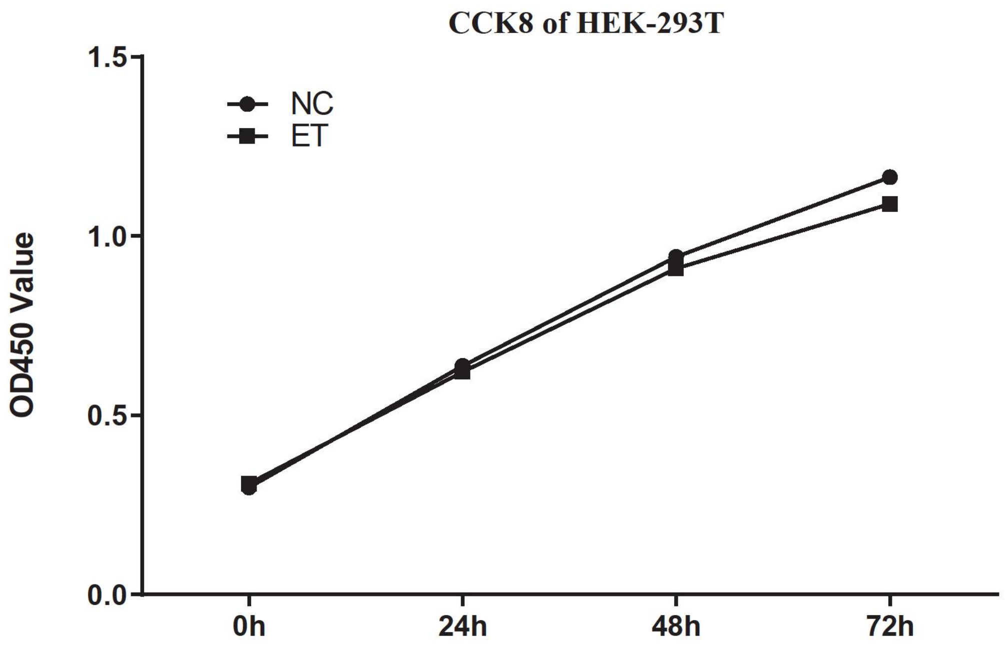


**Figure S6　CCK-8 assay of HEK-293T cells after treatment with Huaier exosome-like nanoparticles (ELNs).**

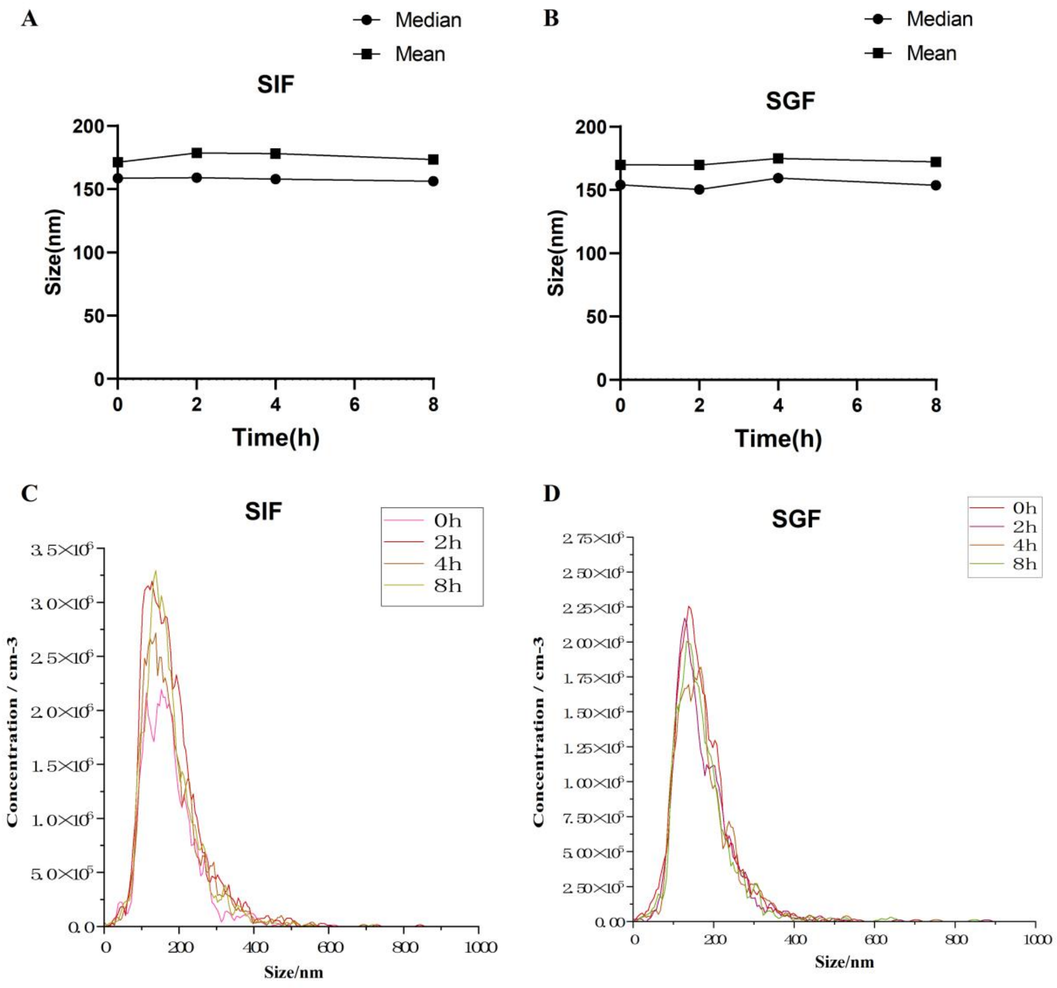


**Figure S7 Stability evaluation of Huaier exosome-like nanoparticles (ELNs) in simulated gastrointestinal fluids.** (A) and (B) Changes in mean particle size and median particle size. After incubating ELNs with simulated intestinal fluid (SIF) and simulated gastric fluid (SGF) at 37°C for 0, 2, 4, and 8 hours, particle size changes were determined by nanoparticle tracking analysis (NTA). (C) and (D) Particle size distribution. Size distribution profiles of ELNs in SIF (C) and SGF (D) after 8 hours of incubation.
